# Accuracy of Pulse Oximeters in Detecting Hypoxemia in Patients with Chronic Thromboembolic Pulmonary Hypertension

**DOI:** 10.1371/journal.pone.0126979

**Published:** 2015-05-15

**Authors:** Tomoki Kohyama, Kiyoshi Moriyama, Riichiro Kanai, Mariko Kotani, Kohji Uzawa, Toru Satoh, Tomoko Yorozu

**Affiliations:** 1 Department of Anesthesiology, Kyorin University School of Medicine, Tokyo, Japan; 2 Division of Cardiology, Second Department of Internal Medicine, Kyorin University School of Medicine, Tokyo, Japan; Nippon Medical School Graduate School of Medicine, JAPAN

## Abstract

**Purpose:**

Pulse oximetry is routinely used to continuously and non-invasively monitor arterial oxygen saturation (SaO_2_). When oxygen saturation by pulse oximeter (SpO_2_) overestimates SaO_2_, hypoxemia may be overlooked. We compared the SpO_2_ - SaO_2_ differences among three pulse oximeters in patients with chronic thromboembolic pulmonary hypertension (CTEPH) who spent their daily lives in a poor oxygen state.

**Material and Method:**

This prospective observational study recruited 32 patients with CTEPH undergoing elective cardiac catheterization. As we collected arterial blood samples in the catheter laboratory, SpO_2_ values were simultaneously recorded. Three pulse oximeters were used on each patient, and SpO_2_ values were compared with oximetry readings using a blood gas analyzer. To determine the optimal SpO_2_ value by which to detect hypoxemia (SaO_2_≦90%), we generated receiver operating characteristic (ROC) curves for each pulse oximeter.

**Result:**

The root mean square of each pulse oximeter was 1.79 (OLV-3100), 1.64 (N-BS), and 2.50 (Masimo Radical). The mean bias (SpO_2_ - SaO_2_) for the 90%–95% saturation range was significantly higher for Masimo Radical (0.19 +/- 1.78% [OLV-3100], 0.18 +/- 1.63% [N-BS], and 1.61 +/- 1.91% [Masimo Radical]; p<0.0001). The optimal SpO_2_ value to detect hypoxemia (SaO_2_≦90%) was 89% for OLV-3100, 90% for N-BS, and 92% for Masimo Radical.

**Conclusion:**

We found that the biases and precision with which to detect hypoxemia differed among the three pulse oximeters. To avoid hypoxemia, the optimal SpO_2_ should be determined for each pulse oximeter.

## Introduction

Pulse oximeters measure oxyhemoglobin saturation (SpO_2_). SpO_2_ is routinely used worldwide not only in the intensive care unit (ICU) [1] and operating room, but also in outpatients [2] to detect patients at risk for hypoxemia. Because a reduction of F_I_O_2_ benefits patients with respiratory failure, it is recommended to maintain a SpO_2_ in mechanically-ventilated patients at approximately 90% [3]. The mechanical ventilation protocol summary by the ARDS network states that the oxygenation goal of patients with ARDS is 55–80 mmHg of PaO_2_ or 88–95% of SpO_2_.

Because such recent oxygen therapies substitute the SpO_2_ for SaO_2_, the accuracy of pulse oximeters around 90% is crucial to avoid hypoxemia, but some studies suggest that SpO_2_ overestimates SaO_2_, especially in patients with critically illnesses. [4] [5]. In a retrospective study including patients with septic shock, Wilson et al. [6] reported that the mean bias (SpO_2_-SaO_2_) was positive and 2.75 +/- 3.1%. Wilson et al. [6] also showed that among patients with 90%-93% SpO_2_ value, 50% of patients were with hypoxemia (SaO_2_< = 90%). Jubran et al. [7] retrospectively evaluated patients in the ICU and found that the cut-off value of SpO_2_ to detect hypoxemia (SaO_2_< = 90%) should be 94%. These results alert the possibility that SpO_2_ overestimates SaO_2_ in the ICU, and a cut-off value of SpO_2_< = 90% may leave patients at risk for hypoxemia.

Because each pulse oximeter follows different algorithms, it is necessary to define optimal SpO_2_ values to avoid hypoxemia by gathering SaO_2_ and SpO_2_ data prospectively from patients in the ICU. Of note, patients in the ICU have different backgrounds and frequently have hemodynamic instability and hypoxemia. Studies have shown that these factors influence the SpO_2_ values [6] [8]. Therefore, patients with poor oxygenation and without hemodynamic instability, hypercapnia and acidosis may be ideal candidates for defining optimal SpO_2_ values to avoid hypoxemia.

In the current study we hypothesized that the accuracy of pulse oximeters can be evaluated in hypoxic patients without hemodynamic instability. For this purpose we recruited patients with chronic thromboembolic pulmonary hypertension (CTEPH) because these patients were considered to live with poor oxygenation. When patients with CTEPH underwent elective cardiac catheterization, we attached 3 different pulse oximeters on their fingers. We measured SpO_2_ using three pulse oximeters and SaO_2_ simultaneously, and calculated biases (SpO_2_-SaO_2_ differences).

## Materials & Methods

### Study Design and Data Collection

This prospective observational study was conducted at Kyorin University Hospital in Tokyo, Japan. This study protocol was approved by our Institutional Review Board on Human Research (number H25-028). Written informed consent of this study was obtained from all patients. The study period was between September 2013 and February 2014. Thirty-two patients with CTEPH who underwent elective cardiac catheterization were recruited in this study.

When right heart catheterization and percutaneous transluminal pulmonary angioplasty [9] are performed in the catheterization laboratory, we routinely monitor SpO_2_ and SaO_2_ in all patients. For the current study, to compare the accuracy of different pulse oximeters, we used three pulse oximeters from three different companies, as follows: OLV-3100 (Nihonkohden, Nishiochiai, Tokyo, Japan); N-BS (Nellcor Puritan Bennett, Pleasonton, CA, USA); and Masimo Radical (Masimo, Irvine, CA, USA). The finger probes used were TL-273T3 (Nihonkohden), D-25 (Nellcor Puritan Bennett), and LNOP Neo-L (Masimo Radical). Each patient was randomly mounted with a total of 3 finger probes from each pulse oximeter on the 2nd, 3rd, and 4th fingers.

The arterial catheter was placed into the radial artery ipsilateral to the pulse oximeter probe. Arterial blood samples were collected anaerobically through the arterial line when patient’s condition was stable and SpO_2_ values were stable for > 30 seconds. Each SpO_2_ value was recorded simultaneously as we collected blood samples. The functional SaO_2_ (HbO_2_ / [hemoglobin+HbO_2_]) was determined using a blood gas analyzer (ABL 825; Radiometer, Copenhagen, Denmark). Fractional SaO_2_ was calculated from the functional SaO_2_ and the measured levels of carboxyhemoglobin and methemoglobin. Quality control standards were run each day. We tried to obtain 3 arterial blood samples from each patient at different time points.

In addition to patients with CTEPH, we enrolled 5 healthy volunteers to collect control data. Under room air condition, 5 healthy volunteers were mounted with 3 finger probes from 3 pulse oximeters, and arterial blood samples were collected anaerobically from their radial artery.

### Statistical Analysis

Assuming an SD of SpO_2_ to be 2 to 3%, we estimated that a sample of 30 patients would need to be enrolled in order for the study to have 90% power, at a two-tailed significance level of 0.05, to detect a mean between-group difference of 1 SD. Data are expressed as the mean +/- standard deviation (SD) and root-mean-square (RMS), as the international standard of pulse oximeter equipment (ISO 9919) states the accuracy of the pulse oximeter equipment in terms of the RMS difference [10]. Data were also analyzed in 3 subgroups of SaO_2_ (85%<SaO2≦90%, 90%<SpO2≦95%, and 95%<SaO2≦100%). Statistical analysis was performed by one-way analysis of variance followed by Tukey’s test. To assess agreement between SpO_2_ and SaO_2_, a Bland-Altman plot was created, and the mean difference as bias, SD as precision, and the 2SD of difference as 95% limits of agreement were calculated. To assess the accuracy of each pulse oximeter to detect hypoxemia, the sensitivity, specificity, and positive and negative predictive values (PPV and NPV) were calculated. Receiver operating characteristic (ROC) curves were constructed. The closest value to the best specificity and sensitivity point on the ROC curve was identified, and the optimal SpO_2_ value for each pulse oximeter was determined. All statistical analyses were performed with EZR (Saitama Medical Center, Jichi Medical University, Omiya, Japan) which is a graphical user interface for R (The R Foundation for Statistical Computing, version 3.1.1) [11]. The levels of significance were set at a p = 0.05.

## Results

### Patient characteristics

**Fig 1:**
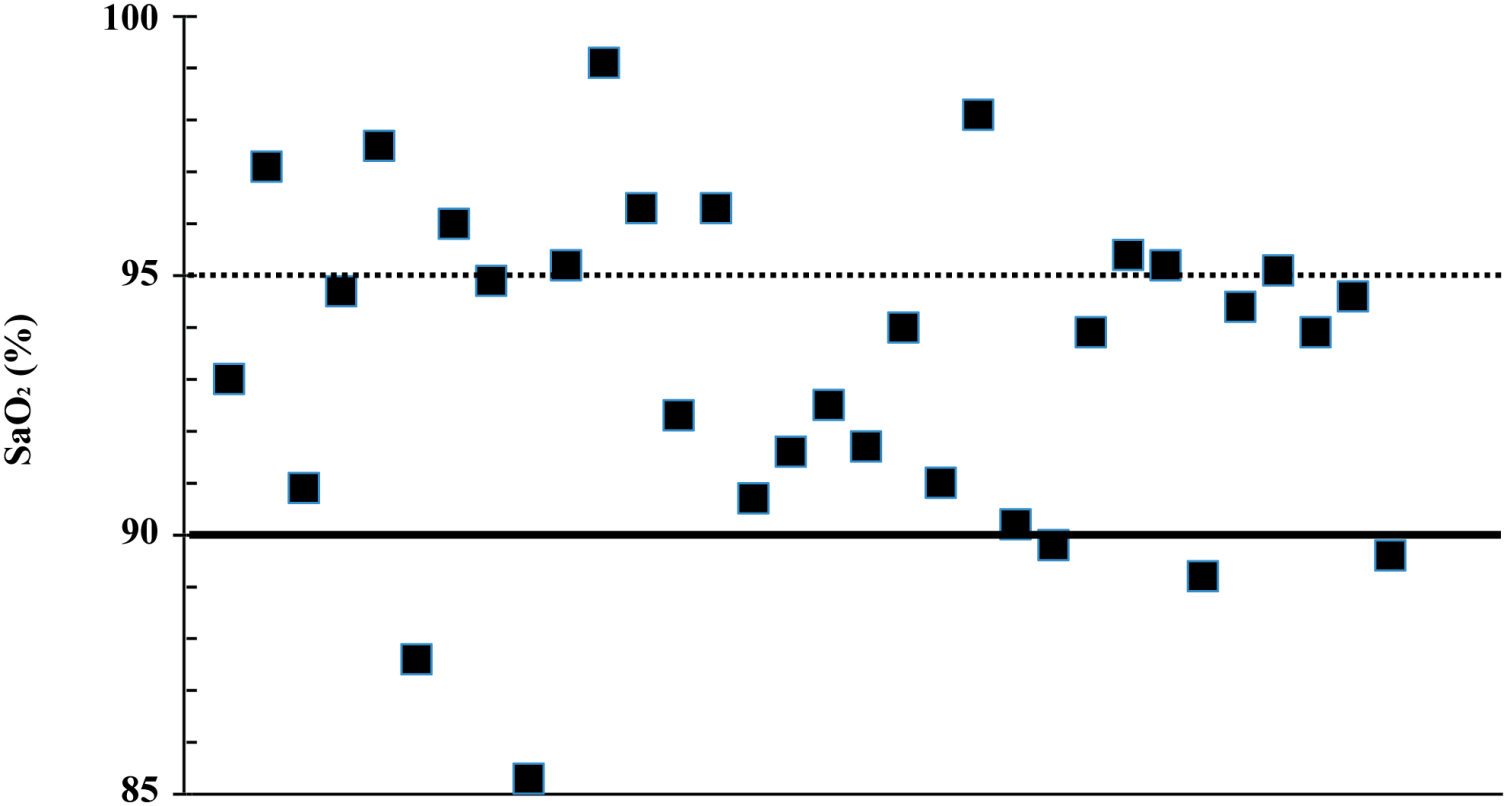
SaO_2_ (oxyhemoglobin saturation) data of 32 patients obtained immediately after arterial catheter placement. Before right heart catheterization, each patient had an arterial catheter placed into their radial artery ipsilateral to the pulse oximeter probes. Arterial blood was sampled, and the SaO_**2**_ was measured by blood gas analyzer (ABL 825). Closed squares indicate initial SaO_**2**_ values of 32 patients enrolled in this study.

We enrolled 32 patients in this study. Table 1 shows the demographics and results of blood gas sampling obtained immediately after arterial catheter placement. Although we tried to obtain three SpO_2_ data by three pulse oximeters at the same time when we collected blood samples, we failed to collect some SpO_2_ values. Therefore, we obtained 92 arterial blood samples, 88 SpO_2_ data points by OLV-3100 (Nihonkohden), 80 data points by N-BS (Nellcor), and 75 data points by Masimo Radical (Masimo). At the beginning of right heart catheterization, the mean values with SD were 93.1% +/-3.5% for SaO_2_. Fig 1 represents the SaO_2_ data obtained immediately after arterial catheter placement. Of 32 patients with CTEPH, 21 (65.6%) had an initial SaO_2_ < = 95%.

**Table 1:** Demographics and laboratory data of the patients.

Variable	Value
Gender (Male / Female)	8 / 24
Age (years)	62.9 +/- 14.7
Height (cm)	156.5 +/- 8.4
Weight (kg)	56.0 +/- 12.6
SaO_2_ (%)	93.1 +/- 3.5
pH	7.432 +/- 0.024
PaCO_2_ (mmHg)	35.3 +/- 3.4
PaO_2_ (mmHg)	66.4 +/- 12.3
Hb (g/dL)	12.1 +/- 1.8
Fraction of Oxyhemoglobin (%)	90.4 +/- 3.3
Carboxyhemoglobin (%)	1.9 +/- 1.2
Deoxyhemoglobin (%)	6.6 +/- 3.0
Methemoglobin (%)	1.0 +/- 0.1

The demographics and results of blood gas sampling obtained immediately after arterial catheter placement are shown. Data are presented as the means ± SD.

*SaO*_*2*_ oxyhemoglobin saturation measured by blood gas analyzer (ABL 825), *PaO*_*2*_ partial pressure of oxygen in arterial blood, *PaCO*_*2*_ partial pressure of arterial carbon dioxide, *Hb* hemoglobin

### Calculated biases (SpO_2_—SaO_2_)

We collected control data from 5 healthy volunteers. Table 2 summarizes data from healthy controls mounted with 3 finger probes from 3 pulse oximeters under room air condition. In 5 patients tested, all the 3 pulse oximeters qualified RMS≦4 (the ISO 9919).

**Table 2:** Calculated biases between SaO_2_ (oxyhemoglobin saturation measured by blood gas analyzer [ABL 825]) and SpO_2_ (oxyhemoglobin saturation measured by 3 pulse oximeters) among 5 healthy volunteers.

	Nihonkohden OLV-3100	Nellcor N-BS	Masimo Radical
Number of samples	5	5	5
SpO_2_—SaO_2_	0.54 +/- 1.14	0.74 +/- 1.55	1.70 +/- 1.41
RMS of (SpO_2_—SaO_2_)	1.16	1.56	1.70

The calculated mean biases (SpO2—SaO2) measured by 3 pulse oximeters are shown. Data are presented as the mean ± SD.

**Fig 2:**
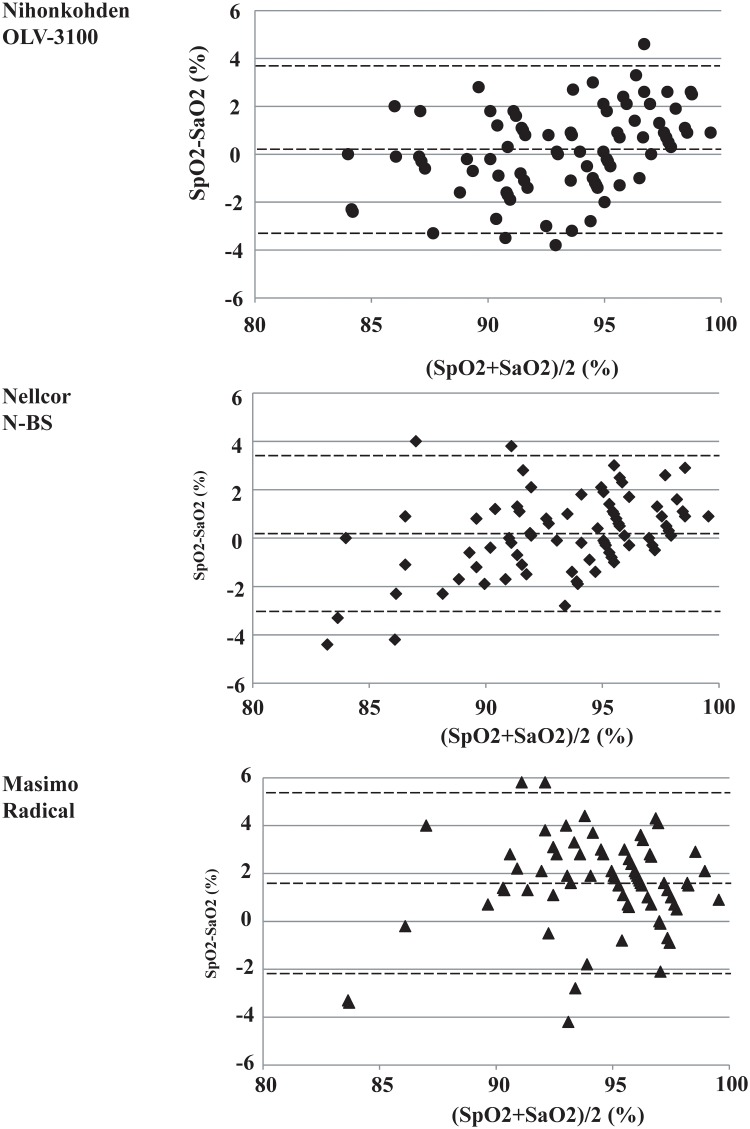
Bland Altman plot comparing SaO_2_ (oxyhemoglobin saturation measured by blood gas analyzer (ABL 825)) and SpO_2_ (oxyhemoglobin saturation measured by 3 pulse oximeters). For each data point, the mean value ([SpO_**2**_ +SaO_**2**_]/ 2) is presented on the x-axis, and the difference value (SpO_**2**_—SaO_**2**_) on the y-axis. *Black lines* represent the 95% confidence interval for SpO_**2**_ [bias ± 2 standard deviation (SD)]. The mean difference value (SpO_**2**_—SaO_**2**_) represents the bias, and SD represents the precision. Oximeters are: A. Nihonkohden OLV-3100, B. Nellcor N-BS, and C. Masimo Radical. Bias was 0.19% +/- 1.79% (mean +/- SD) by Nihonkohden oximeter, 0.18% +/- 1.64% by Nellcor oximeter, and 1.61% +/- 1.93% by Masimo oximeter.

Table 3 shows the calculated mean biases (SpO_2_—SaO_2_) measured by 3 pulse oximeters for the 85%–100% saturation range. All three pulse oximeters showed positive biases, suggesting that SpO_2_ overestimated SaO_2_. The mean bias by Masimo Radical was significantly higher than the mean bias by Nihonkohden OLV-3100 and Nellcor N-BS (P<0.0001). Although all 3 pulse oximeters qualified for the ISO 9919 (RMS≦4); the RMS by Masimo Radical had the highest value. Fig 2 is the Bland Altman plot indicating a bias and limits of agreement.

**Table 3:** Calculated biases between SaO_2_ (oxyhemoglobin saturation measured by blood gas analyzer [ABL 825]) and SpO_2_ (oxyhemoglobin saturation measured by 3 pulse oximeters).

	Nihonkohden OLV-3100	Nellcor N-BS	Masimo Radical
Number of samples	88	80	75
SpO_2_—SaO_2_	0.19 +/- 1.78	0.18 +/- 1.63	1.61 +/- 1.91*
RMS of (SpO_2_—SaO_2_)	1.79	1.64	2.50

The calculated mean biases (SpO2—SaO2) measured by 3 pulse oximeters for the 85%–100% saturation range are shown. Data are presented as the mean ± SD and analyzed by one-way analysis of variance followed by Tukey’s test.

*p<0.0001 vs. Nihonkohden OLV-3100 and Nellcor N-BS

Some patients had oxygen administered to avoid hypoxemia during the procedure. Actually, 35/92 blood samples were collected from patients with oxygen inhalation. Table 4 shows data from patients under room air condition.

**Table 4:** Calculated biases between SaO_2_ (oxyhemoglobin saturation measured by blood gas analyzer [ABL 825]) and SpO_2_ (oxyhemoglobin saturation measured by 3 pulse oximeters) among patients under room air condition.

	Nihonkohden OLV-3100	Nellcor N-BS	Masimo Radical
Number of samples	54	50	51
SpO_2_—SaO_2_	0.15 +/- 1.94	0.05 +/- 1.95	1.55 +/- 1.55*
RMS of (SpO_2_—SaO_2_)	1.93	1.83	2.63

The calculated mean biases (SpO2—SaO2) measured by 3 pulse oximeters for the 85%–100% saturation range are shown. Data are presented as the mean ± SD and analyzed by one-way analysis of variance followed by Tukey’s test.

*p<0.0001 vs. Nihonkohden OLV-3100 and Nellcor N-BS

### Subgroup analysis

**Fig 3:**
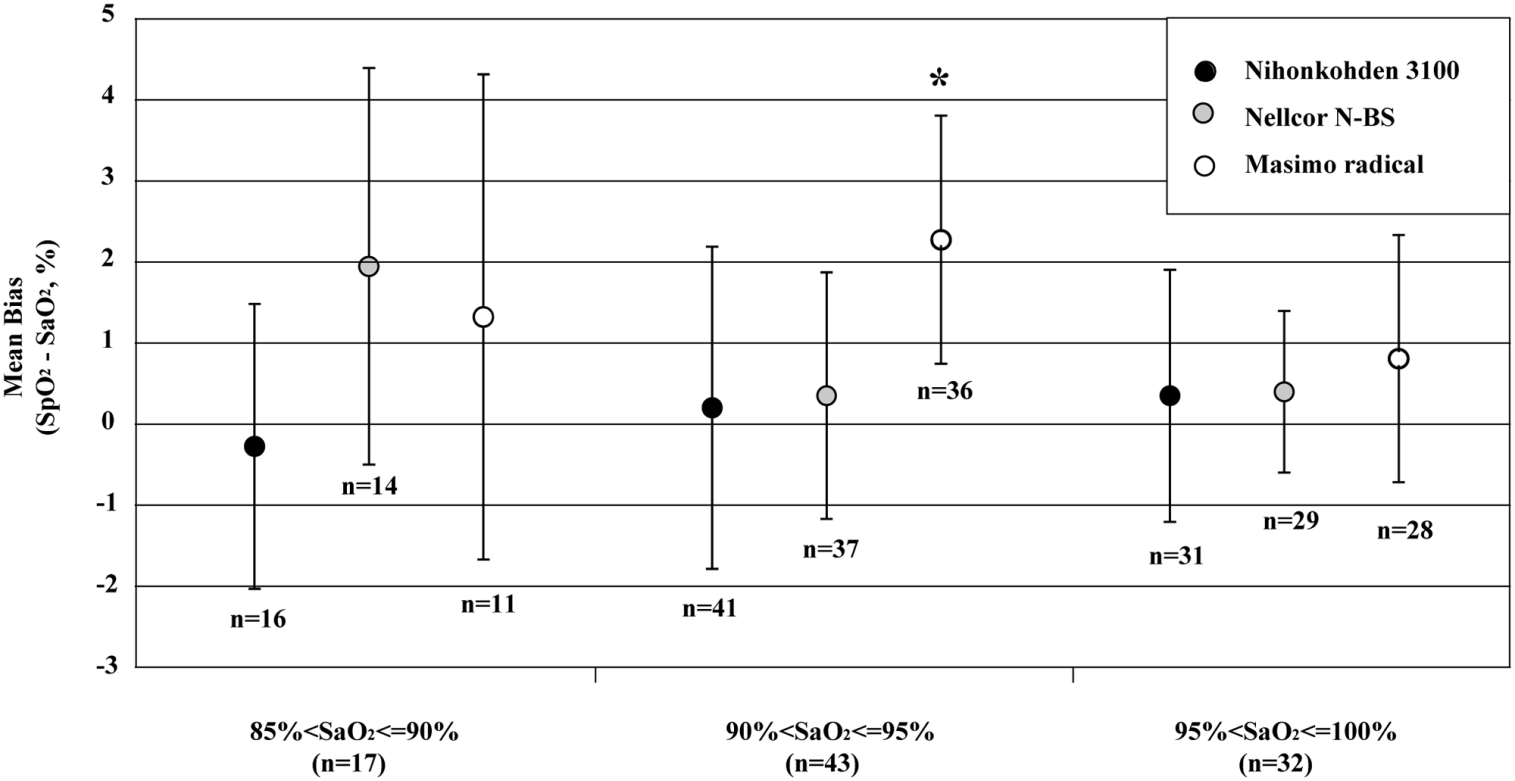
Bias (mean ± SD) for the 3 oximeters in the 5% range of SaO_2_ (oxyhemoglobin saturation measured by blood gas analyzer [ABL 825]). Bias is calculated as SpO_**2**_ (oximeter-measured value of oxyhemoglobin saturation) minus SaO_**2**_. SpO_**2**_ measured by Nihonkohden 3100 are indicated with closed circles, SpO_**2**_ by Nellcor N-BS with gray circles, and SpO_**2**_ by Masimo radical with open circles. Data are presented as the mean ± SD and analyzed by one-way analysis of variance followed by Tukey’s test: * p<0.0001 vs. Nihonkohden 3100 and Nellcor N-BS.

We questioned whether or not the biases of pulse oximeters are influenced by the SaO_2_ range. Fig 3 shows the calculated biases (SpO_2_—SaO_2_) in the 5% range of SaO_2_. Because the number of samples was small (n = 17), SD values tended to be higher in the 85%< SaO_2_≦90% range. The mean bias by the Masimo Radical for the 90%< SpO_2_≦95% saturation range was significantly higher compared with the mean bias by the Nihonkohden OLV-3100 and Nellcor N-BS (0.19 +/- 1.99 for Nihonkohden OLV-3100, 0.34 +/- 1.52 for Nellcor N-BS, and 2.26 +/- 1.53 for Masimo Radical; p<0.0001 vs. Nihonkohden 3100 and Nellcor N-BS). There were no significant differences in the other 2 ranges (85%< SpO_2_≦90% and 95%< SpO_2_≦100% saturation range).

### Cut-off value of SpO_2_

We hypothesized that the cut-off value of each pulse oximeter to detect hypoxemia (SaO_2_< = 90% or PaO_2_< = 60 mmHg) was different. We calculated the sensitivity, specificity, PPV, and NPV of each pulse oximeter to detect hypoxemia (Table 5 and Table 6). As shown in Table 5, the specificity ([patients with SaO_2_>90 and SpO_2_>90] / [patients with SaO_2_>90]) by Masimo Radical was 45.5% and the NPV ([patients with SaO_2_>90 and SpO_2_>90] / [patients with SpO_2_>90]) by Nihonkohden 3100 was 65.0%.

**Table 5:** Optimal SpO_2_ value and ability of 3 pulse oximeters to detect hypoxemia (SaO_2_≦90%).

	Cut-off value of SpO_2_ (%)	Sensitivity (%)	Specificity (%)	PPV (%)	NPV (%)	Accuracy (%)
Nihon-kohden OLV-3100	90	90.3	81.2	95.6	65.0	88.6
Nellcor N-BS	90	93.9	85.7	96.9	75.0	92.5
Masimo Radicalp	90	100	45.5	91.4	100	92.0
	Optimal SpO_2_ (%)^#^					
Nihon- kohden OLV-3100	89	97.2	81.2	95.9	86.7	94.3
Nellcor N-BS	90	93.9	85.7	96.9	75.0	92.5
Masimo Radical	92	93.8	81.8	96.8	69.2	92.0

The sensitivity, specificity, positive predictive values, and negative predictive values of each pulse oximeter to detect hypoxemia are shown with the calculated optimal SpO_2_ value to detect hypoxemia.

*PPV* positive predictive values, *NPV* negative predictive values

Sensitivity = (patients with SaO_2_< = 90 and SpO_2_< = 90) / (patients with SaO_2_< = 90)

Specificity = (patients with SaO_2_>90 and SpO_2_>90) / (patients with SaO_2_>90)

PPV = (patients with SaO_2_< = 90 and SpO_2_< = 90) / (patients with SpO_2_< = 90)

NPV = (patients with SaO_2_>90 and SpO_2_>90) / (patients with SpO_2_>90)

^#^Optimal SpO_2_ represents the best compromise between sensitivity and specificity to detect hypoxemia (SaO2≦90%)

**Table 6:** Optimal SpO_2_ value and ability of 3 pulse oximeters to detect hypoxemia (PaO_2_≦60 mmHg).

	Cut-off value of SpO_2_ (%)	Sensitivity (%)	Specificity (%)	PPV (%)	NPV (%)	Accuracy (%)
Nihon Kohden OLV-3100	90	98.2	61.3	82.4	95.0	85.2
Nellcor N-BS	90	100	57.1	81.2	100	85.0
Masimo Radical	90	100	23.8	77.1	100	78.7
	Optimal SpO_2_ (%)^#^					
Nihon Kohden OLV-3100	92	86.0	96.8	98.0	78.9	89.8
Nellcor N-BS	93	82.7	100	100	75.7	88.8
Masimo Radical	95	97.8	95.2	97.8	95.2	97.0

The sensitivity, specificity, positive predictive values, and negative predictive values of each pulse oximeter to detect hypoxemia are shown with the calculated optimal SpO_2_ value to detect hypoxemia.

*PPV* positive predictive values, *NPV* negative predictive values

Sensitivity = (patients with PaO_2_< = 60 and SpO_2_< = 90) / (patients with PaO_2_< = 60)

Specificity = (patients with PaO_2_>60 and SpO_2_>90) / (patients with PaO_2_>60)

PPV = (patients with PaO_2_< = 60 and SpO_2_< = 90) / (patients with SpO_2_< = 90)

NPV = (patients with PaO_2_>60 and SpO_2_>90) / (patients with SpO_2_>90)

^#^Optimal SpO_2_ represents the best compromise between sensitivity and specificity to detect hypoxemia (PaO_2_≦60 mmHg)

Next, we calculated the optimal SpO_2_ value to detect hypoxemia by constructing ROC curves. The optimal SpO_2_ value was 89% for Nihonkohden 3100, 90% for Nellcor N-BS, and 92% for Masimo Radical. Using the optimal SpO_2_ value of each pulse oximeter, the sensitivity, specificity, PPV, and NPV were re-calculated. The specificity by Masimo Radical increased from 45.5% to 81.8%, although the NPV decreased from 100% to 69.2%. The NPV by Nihonkohden 3100 increased from 65.0% to 86.7%, and the sensitivity also increased from 90.3% to 97.2%.

## Discussion

### Key findings

In this study we evaluated the accuracy of three pulse oximeters to detect hypoxemia in patients with CTEPH who spend their daily life in a poor oxygen state. Among 32 patients enrolled in this study, 21 (65.6%) had a SaO_2_ < = 95%. We found that all three pulse oximeters had positive biases, suggesting that all SpO_2_ values overestimated SaO_2_ values. Among 3 pulse oximeters, significant differences were detected in calculated biases (SpO_2_—SaO_2_), especially in the 90%< SaO_2_≦95% range. We also found that the optimal cut-off values to detect hypoxemia were slightly different among the 3 pulse oximeters (89% for Nihonkohden 3100, 90% for Nellcor N-BS, and 92% for Masimo Radical). Our results suggest that when we substitute SpO_2_ for SaO_2_, the optimal SpO_2_ should be determined for each pulse oximeter to avoid hypoxemia.

### Relationship to previous studies

Several data suggest that SpO_2_ overestimates SaO_2_ in patients with critically illnesses. In an observational prospective study involving patients admitted to the ICU, Van de Louw et al. [1] reported that the accuracy of SpO_2_ appeared to be influenced by the type of oximeter, the presence of hypoxemia, and the requirement for vasoactive drugs. Van de Louw et al. [1] suggested that a SpO2 > 94% appears necessary to ensure a SaO_2_ of 90%. In a retrospective study, Wilson et al. [6] showed that pulse oximetry overestimated the SaO_2_ by a mean of 2.75% in emergency department patients with severe sepsis and septic shock. Wilson et al. [6] found that the overestimation was exacerbated by the presence of hypoxemia. Of ICU patients, Ghayumi et al. [12] showed that a SpO_2_ cut-off value ≦94% could predict hypoxemia (PaO_2_<60 mmHg) with a sensitivity of 100% and a specificity of 95% in liver transplant candidates. The Ghayumi et al. [12] study included patients with a mean SaO_2_ value of 95.19%, in contrast to the 93.1% mean SaO_2_ value in our study, with more stable conditions of patients. Our results are consistent with previous studies that showed SpO_2_ overestimates SaO_2_. (1,6)

### Significance and implications

In this study we put more emphasis on the ability of pulse oximeters to detect hypoxemia rather than RMS differences (SpO_2_-SaO_2_) that is an international standard parameter of the accuracy of SpO_2_. We found that Masimo Radical had 100% of sensitivity and NPV with lowest specificity (Table 5). These statistical parameters suggest that Masimo Radical is most reliable when patients have a SaO_2_< = 90%. In the clinical setting, especially in ICU, the purpose of monitoring SpO_2_ is to avoid hypoxemia, keeping the SaO_2_ > 90%. After applying a calculated optimal SpO_2_ (92% for the Masimo Radical), the specificity increased from 45.5% to 81.8%, while the NPV decreased from 100% to 69.2%. These changes in statistical parameters are more favorable in keeping the SaO_2_ > 90%, and beneficial in the clinical setting. These results imply that when we define a statistical “optimal value” for medical monitors, we must consider the clinical purpose of each monitor.

### Strengths and Limitations

There were several limitations in this study. First, because this study was performed in Japan and only included patients with CTEPH, all of the patients were Asian (Japanese) and 75% of the patients were female. Because Feiner et al. [13] reported that skin color and gender are predictive of errors in SpO_2_ estimates at low SaO_2_ levels (< 80%), our results may not be applicable to patients of general. In addition, some data were gathered during the catheter procedure. These procedures might have affected hemodynamics and resulted in increased biases. Although several factors might have influenced the accuracy of SpO_2_, we speculate that the unified backgrounds and stable conditions of the patients trump these drawbacks; however, further studies are needed including patients with different races.

Second, we put too much emphasis on detecting SaO_2_ values < 90%. SpO_2_ is simply a non-invasive monitor substituting SaO_2_. In the clinical situation, especially in the general ward, clinicians have a tendency to expect a safer SpO_2_ range, not approximately 90%, but > 95%. With that point of view, there were no significant differences among the 3 pulse oximeters in the 95<SaO_2_≦100% range. Thus, keeping patients in the 95<SaO_2_≦100% range, all the pulse oximeters were reliable and there was no need for detecting the optimal SpO_2_. However, recent evidence suggests that conservative oxygen (targeting a SpO_2_ between 90% and 92%) therapy may be beneficial to critically ill patients [14]. Indeed, the accuracy of a SpO_2_ of approximately 90% may be important for future oxygen therapy. We suggest that further studies are warranted to evaluate “optimal” oxygen therapy on the assumption that SpO_2_ overestimates SaO_2_, and optimal SpO_2_ to detect hypoxemia differs among pulse oximeters.

Third, because this study was limited to patients with CTEPH, these results should be limited to normo/or hypocapnic patients, and may not be applicable to patients with hypercapnia, acidosis and hemodynamic instability. As oxygen dissociation curve clearly illustrates, the SaO_2_ sure changes according to PaCO_2_ level and pH. It is also well-known that hemodynamic instability affects the accuracy of pulse oximeters [6]. Further studies are recommended including patients with COPD, who spend their daily life with hypercapnia, and patients with septic shock.

## Conclusions

In conclusion, we found that SpO_2_ measured by 3 pulse oximeters overestimated the SaO_2_, and the optimal cut-off value to detect hypoxia was slightly different among 3 pulse oximeters (89% for Nihonkohden 3100, 90% for Nellcor N-BS, and 92% for Masimo Radical). We suggest that when SpO_2_ is substituted for SaO_2_, optimal SpO_2_ should be determined for each pulse oximeter to avoid hypoxemia.

## References

[1] Van de Louw A, Cracco C, Cerf C, Harf A, Duvaldestin P, Lemaire F, et al (2001) Accuracy of pulse oximetry in the intensive care unit. Intensive Care Med 27:1606–1613 11685301 10.1007/s001340101064

[2] Majumdar SR, Eurich DT, Gamble JM, Senthilselvan A, Marrie TJ (2011) Oxygen saturations less than 92% are associated with major adverse events in outpatients with pneumonia: a population-based cohort study. Clin Infect Dis 52:325–31 10.1093/cid/ciq076 21217179

[3] Vadász I, Sznajder Jl (2010) Update in acute lung injury and critical care. Am J Respir Crit Care Med 183:1147–52 10.1164/rccm.201102-0327UP 21531954 PMC3114050

[4] Perkins GD, McAuley DF, Giles S, Routledge H, Gao F (2003) Do changes in pulse oxymeter oxygen saturation predict equivalent changes in arterial oxygen saturation? Crit Care 7:R67–R71 12930558 10.1186/cc2339 PMC270702

[5] Seguin P. Le Rouzo A, Tanguy M, Guillou YM, Feillu A, Malledant Y (2000) Evidence for the need of bedside accuracy of pulse oximetry in an intensive care unit. Crit Care Med 28:703–706 10752818 10.1097/00003246-200003000-00017

[6] Wilson BJ1, Cowan HJ, Lord JA, Zuege DJ, Zygun DA (2010) The accuracy of pulse oximetry in emergency department patients with severe sepsis and septic shock: a retrospective cohort study. BMC Emerg Med 10:9 10.1186/1471-227X-10-9 20444248 PMC2876142

[7] Jurbran A, Tobin MJ (1990) Reliability of pulse oximetry in titrating supplemental oxygen therapy in ventilator-dependent patients. Chest 97:1420–1425 2347228 10.1378/chest.97.6.1420

[8] Secker C1, Spiers P (1997) Accuracy of pulse oximetry in patients with low systemic vascular resistance. Anaesthesia 52:127–30. 9059094 10.1111/j.1365-2044.1997.32-az0062.x

[9] Kataoka M, Inami T, Hayashida K, Shimura N, Ishiguro H, Abe T, et al (2012) Percutaneous transluminal pulmonary angioplasty for the treatment of chronic thromboembolic pulmonary hypertension. Circ Cardiovasc Interv. 5:756–62. 10.1161/CIRCINTERVENTIONS.112.971390 23132237

[10] Ikeda K, MacLeod DB, Grocott HP, Moretti EW, Ames W, Vacchiano C (2014) The accuracy of a near-infrared spectroscopy cerebral oximetry device and its potential value for estimating jugular venous oxygen saturation. Anesth Analg 119:1381–92 10.1213/ANE.0000000000000463 25313967 PMC4237713

[11] Kanda Y (2012) Investigation of the freely available easy-to-use software 'EZR' for medical statistics. Bone Marrow Transplant 48:452–458 10.1038/bmt.2012.244 23208313 PMC3590441

[12] Ghayumi SM, Khalafi-Nezhad A, Jowkar Z (2014) Pulse oximeter oxygen saturation in prediction of arterial oxygen saturation in liver transplant candidates. Hepat Mon 14:e15449 10.5812/hepatmon.15449 24748894 PMC3989597

[13] Feiner JR1, Severinghaus JW, Bickler PE (2007) Dark skin decreases the accuracy of pulse oximeters at low oxygen saturation: the effects of oximeter probe type and gender. Anesth Analg 105:S18–23 18048893 10.1213/01.ane.0000285988.35174.d9

[14] Suzuki S, Eastwood GM, Glassford NJ, Peck L, Young H, Garcia-Alvarez M, et al Crit Care Med (2014) Conservative oxygen therapy in mechanically ventilated patients: a pilot before-and-after trial. 42:1414–22 10.1097/CCM.0000000000000219 24561566

