# Correction: Accuracy of Pulse Oximeters in Detecting Hypoxemia in Patients with Chronic Thromboembolic Pulmonary Hypertension

**DOI:** 10.1371/journal.pone.0141944

**Published:** 2015-10-28

**Authors:** Tomoki Kohyama, Kiyoshi Moriyama, Riichiro Kanai, Mariko Kotani, Kohji Uzawa, Toru Satoh, Tomoko Yorozu

There is an error in the first three sentences under the subheading “Cut-Off value of SpO_2_” in the Results section. The correct sentences are: We hypothesized that the cut-off value of each pulse oximeter to detect hypoxemia (SaO_2_< = 90% or PaO_2_< = 60 mmHg) was different. We calculated the sensitivity, specificity, PPV, and NPV of each pulse oximeter to detect hypoxemia. As shown in Table 5, the specificity ([patients with SaO_2_< = 90 and SpO_2_< = 90] / [patients with SaO_2_< = 90]) by Masimo Radical was 45.5% and the NPV ([patients with SaO_2_< = 90 and SpO_2_< = 90] / [patients with SpO_2_>90]) by Nihonkohden 3100 was 65.0%.

**Table 5:** Optimal SpO_2_ value and ability of 3 pulse oximeters to detect hypoxemia (SaO_2_≦90%).

	Cut-off value of SpO_2_ (%)	Sensitivity (%)	Specificity (%)	PPV (%)	NPV (%)	Accuracy (%)
Nihon-kohden OLV-3100	90	90.3	81.2	95.6	65.0	88.6
Nellcor N-BS	90	93.9	85.7	96.9	75.0	92.5
Masimo Radicalp	90	100	45.5	91.4	100	92.0
	Optimal SpO_2_ (%)^#^					
Nihon- kohden OLV-3100	89	97.2	81.2	95.9	86.7	94.3
Nellcor N-BS	90	93.9	85.7	96.9	75.0	92.5
Masimo Radical	92	93.8	81.8	96.8	69.2	92.0

The sensitivity, specificity, positive predictive values, and negative predictive values of each pulse oximeter to detect hypoxemia are shown with the calculated optimal SpO_2_ value to detect hypoxemia.

*PPV* positive predictive values, *NPV* negative predictive values

Sensitivity = (patients with SaO_2_>90 and SpO_2_>90) / (patients with SaO_2_>90)

Specificity = (patients with SaO_2_< = 90 and SpO_2_< = 90) / (patients with SaO_2_< = 90)

PPV = (patients with SaO_2_>90 and SpO_2_>90) / (patients with SpO_2_>90)

NPV = (patients with SaO_2_< = 90 and SpO_2_< = 90) / (patients with SpO_2_< = 90)

^#^Optimal SpO_2_ represents the best compromise between sensitivity and specificity to detect hypoxemia (SaO_2_≦90%)

There are also errors in the footnotes of Tables 5 and 6. Please view the corrected Tables 5 and 6 here.

**Table 6:** Optimal SpO_2_ value and ability of 3 pulse oximeters to detect hypoxemia (PaO2≦60 mmHg).

	Cut-off value of SpO_2_ (%)	Sensitivity (%)	Specificity (%)	PPV (%)	NPV (%)	Accuracy (%)
Nihon Kohden OLV-3100	90	98.2	61.3	82.4	95.0	85.2
Nellcor N-BS	90	100	57.1	81.2	100	85.0
Masimo Radical	90	100	23.8	77.1	100	78.7
	Optimal SpO_2_ (%)^#^					
Nihon Kohden OLV-3100	92	86.0	96.8	98.0	78.9	89.8
Nellcor N-BS	93	82.7	100	100	75.7	88.8
Masimo Radical	95	97.8	95.2	97.8	95.2	97.0

The sensitivity, specificity, positive predictive values, and negative predictive values of each pulse oximeter to detect hypoxemia are shown with the calculated optimal SpO2 value to detect hypoxemia.

PPV positive predictive values, NPV negative predictive values

Sensitivity = (patients with PaO_2_>60 and SpO_2_>90) / (patients with PaO_2_>60)

Specificity = (patients with PaO_2_< = 60 and SpO_2_< = 90) / (patients with PaO_2_< = 60)

PPV = (patients with PaO_2_>60 and SpO_2_>90) / (patients with SpO_2_>90)

NPV = (patients with PaO_2_< = 60 and SpO_2_< = 90) / (patients with SpO_2_< = 90)

^#^Optimal SpO_2_ represents the best compromise between sensitivity and specificity to detect hypoxemia (PaO_2_≦60 mmHg)
